# Starling Medical College

**Published:** 1848-04

**Authors:** 


*Starling Medical College.*—The Professors, medical class, and friends of this Institution lately celebrated the organization, under the new charter and title, by a sumptuous supper. Speeches were made, sentiments offered, and altogether, if we may judge from the report in the *Daily Ohio State Journal*, the occasion was a joyous one.

The veteran Prof. Childs presided. We cannot refrain from quoting the report of his remarks, which, as coming from a Massachusetts professor who has seen something of the West. and of Western Institutions, are relevant to a discussion in the original department of the present No. of this Journal:—

"After the good things were despatched, the President for the evening, the venerable Professor Childs, of Massachusetts, made a very vigorous and animated speech. He dwelt particularly upon the destiny and prospects of the great West, and contended that our mental and physical energies were developing here more rapidly than in any other part of our country. There seemed to be more room for expansion, a wider field for enterprize, and a greater freshness and vigor in everything, than could be found elsewhere. It was scarcely possible to conceive of the destiny that awaited us, if we were true to our own best interests."

His excellency Gov. Bebb, and other distinguished functionaries of the Buckeye State were among the invited guests. Success to the Starling Medical College! We trust that other medical colleges may find *Starlings*, or, at least, *birds of the same feather*.

